# The role of community health workers in the surgical cascade: a scoping review

**DOI:** 10.1186/s12960-021-00659-z

**Published:** 2021-10-03

**Authors:** Helen W. Li, Michael L. Scanlon, Nicholas Kisilu, Debra K. Litzelman

**Affiliations:** 1 Department of Surgery, Washington University School of Medicine in St Louis, St. Louis, MO United States of America; 2 Indiana University Center for Global Health, 702 Rotary Circle, Suite RO 101, Indianapolis, IN 46202 United States of America; 3 Department of General Surgery and Anesthesiology, Moi University School of Medicine, Eldoret, Kenya; 4 William M. Tierney Center for Health Services Research, Regenstrief Institute, Inc. and Indiana University School of Medicine, 1101 West 10th Street, Indianapolis, IN 46202 United States of America

**Keywords:** Community health workers, CHW, Surgery, Surgical cascade, Outcomes

## Abstract

**Background:**

Community health workers (CHWs) can increase access to various primary healthcare services; however, their potential for improving surgical care is under-explored. We sought to assess the role of CHWs in the surgical cascade, defined as disease screening, linkage to operative care, and post-operative care. Given the well-described literature on CHWs and screening, we focused on the latter two steps of the surgical cascade.

**Methods:**

We conducted a scoping review of the peer-reviewed literature. We searched for studies published in any language from January 1, 2000 to May 1, 2020 using electronic literature databases including Pubmed/MEDLINE, Web of Science, SCOPUS, and Google Scholar. We included articles on CHW involvement in linkage to operative care and/or post-operative surgical care. Narrative and descriptive methods were used to analyze the data.

**Results:**

The initial search identified 145 articles relevant to steps in the surgical cascade. Ten studies met our inclusion criteria and were included for review. In linkage to care, CHWs helped increase surgical enrollment, provide resources for vulnerable patients, and build trust in healthcare services. Post-operatively, CHWs acted as effective monitors for surgical-site infections and provided socially isolated patients with support and linkage to additional services. The complex and wide-ranging needs of surgical patients illustrated the need to view surgical care as a continuum rather than a singular operative event.

**Conclusion:**

While the current literature is limited, CHWs were able to maneuver complex medical, cultural, and social barriers to surgical care by linking patients to counseling, education, and community resources, as well as post-operative infection prevention services. Future studies would benefit from more rigorous study designs and larger sample sizes to further elucidate the role CHWs can serve in the surgical cascade.

## Introduction

The *Lancet Commission on Global Surgery* estimates that 5 billion people lack access to safe, affordable surgery [1]. Recent estimates show 30% of global disease burden is surgical. Rising rates of non-communicable disease, including cancer, heart disease and diabetes, often amenable to surgery, now have mortality rates double that of infectious diseases, maternal and perinatal conditions, and malnutrition [2–5]. This increased surgical burden is exacerbated by the global shortage of surgeons, anaesthesiologists, and obstetricians [4, 6, 7].

In many low and middle-income countries (LMICs), surgical conditions including appendicitis, fractures, hernias, obstructed labor, and breast and cervical cancer lead to high morbidity and mortality. One important factor in this disparity is an insufficient surgical capacity, as 19% of the world’s surgeons serve 48% of the world’s population. Of these surgeons, a disproportionate number are concentrated in urban areas and practice in a fee-for-service model, further exacerbating the true shortage of the surgical workforce available to underserved communities [8].

Surgical workforce inadequacies are also seen in high-income countries (HICs), particularly in rural communities. Australia, Canada, South Africa, and the US have initiated rural surgery residency tracks in an effort to increase the much needed population of rural surgeons [9]. In 2009, nearly 4.6 million people were reported to be without access to emergency surgical treatment in the US, which has been worsened by waves of rural critical access hospital closures, creating “surgical deserts” in many parts of the country [6, 10–12].

Despite the World Health Organization’s push for strengthening emergency and essential surgical care as a component of Universal Health Coverage, surgical care has traditionally received low priority on global health agendas [13]. High out-of-pocket payments and low insurance enrolment in many health systems leave catastrophic costs for patients, while inadequate surgical capacity may result in a complete absence of surgical care in underserved areas [14]. There is an urgent need to identify interventions to expand access to quality surgical care across the surgical cascade, defined as a three-step process, including screening, linkage to operative management, and post-operative follow-up care [15].

Community health workers (CHWs) are key members of health systems and have been central to many countries’ efforts to expand primary healthcare services. According to the CHW Section of the American Public Health Association, a CHW is “a frontline public health worker who is a trusted member of and/or has an unusually close understanding of the community served. This trusting relationship enables the worker to serve as a liaison, link, or intermediary between health and social services and the community to facility access to services and improve the quality and cultural competence of service delivery” [16]. Involvement of CHWs have been shown to effectively improve screening, linkage to care, retention in care, and healthcare access and outcomes within many areas of primary health care [17, 18]. CHWs may play a vital role within surgical care as well.

To date, the majority of CHW work in surgical disease has origins in oncology [19]. A substantial body of literature addresses CHW involvement in screening for colorectal, breast, and cervical cancers. Within this realm, CHWs have played a valuable role in educating and connecting patients, particularly in vulnerable communities, to screening services for treatable and preventable cancers, overcoming difficult cultural and educational barriers often associated with sensitive screening exams [20–26]. However, their potential for improving access to surgical care and outcomes beyond the first step of disease screening is under-explored [27]. We conducted a scoping review of the published literature on the role and impact of CHWs within the latter two steps of the surgical cascade, namely, linkage to care and post-operative care.

## Methods

We searched for recent literature published in any language from January 1, 2000 to May 1, 2020 in Pubmed/MEDLINE, Web of Science, SCOPUS, and Google Scholar. We followed reporting guidelines as outlined in the Preferred Reporting Items for Systematic review and Meta-Analysis extension for Scoping Reviews (PRISMA-ScR) [28].

One author (HWL) implemented the search strategy to retrieve the initial list of titles from each database. The search strategy combined relevant terms using Boolean operators to search titles, abstracts, keywords, and subject headings. Two overarching “concept” terms were identified— “community health worker” (concept term #1) and “surgery” (concept term #2)—that each included approximately ten related terms that were used in our search strategy. The basic logic of the search strategy was: [Terms under concept #1 connected by ‘OR’] ‘AND’ [Terms under concept #2 connected with ‘OR’]. Specific search terms are provided in Table 1.

**Table 1:** Study search strategy

	Keywords used
Concept 1: Community health worker	“community health worker,” “community health volunteer,” “peer navigator,” “paraprofessional,” “outreach worker,” “lay advocate,” “community health advisor,” “lay health worker,” and “lay health advisor”
Concept 2: Surgery	“surgery,” “surgical,” “operation,” “operative,” “general surgery,” “surgical oncology,” “otolaryngology,” “ophthalmology,” “colorectal,” “urology,” “neurosurgery,” “orthognathic surgery,” “orthopedic surgery,” “plastic surgery,” “thoracic surgery,” “cardiovascular surgery,” “obstetrical surgery,” “trauma surgery,” and “traumatology”

We also used relevant Medical Subject Headings (MeSH) terms to search PubMed/MEDLINE and SCOPUS that employ MeSH terms as part of their controlled vocabulary. Retrieved titles from each database were imported into separate EndNote (Version X9, Clarivate Analytics) libraries and then combined into a master library. Duplicates were removed using the EndNote duplicate function as well as manual review of title names. Two authors (HWL and MLS) independently reviewed titles, abstracts, and full texts. At each stage—title review, abstract review, and full text review—the authors compared included and excluded articles and resolved discrepancies through discussion and consultation with a third author. We also searched the bibliographies of all studies included in our review for additional relevant articles.

We included review articles, descriptive studies, interventional studies, and study protocols published in peer-reviewed journals. Commentaries, editorials, letters to the editor, and dissertations were excluded. To be included, articles reported on a community-based program, intervention, or policy that included CHW activities related to the latter two steps of the surgical cascade, involving linkage to care for surgery-related services and/or post-operative surgical care. We defined CHWs as a paid or unpaid individual who provides health-related services in non-facility-based settings to a specific community of which they typically are a member. Articles did not have to use the term “community health worker” as long as they provided sufficient information on their role for the authors’ to determine that they met this definition and that their activities were community, and not solely facility, based.

Among studies included for review, one author (HWL) extracted data on each study’s design, location and population, outcomes measured, and findings using a structured extraction guide as previously agreed on by all authors (Table 2). We also classified the surgical specialty and surgical cascade stage examined in each study and the role and training of CHWs involved, if available. Extracted data were organized in a database using Microsoft Excel. Two authors (HWL and MLS) used narrative and descriptive methods to analyze the data.

**Table 2:** Characteristics and findings of included studies

Author (year)	Study location	Study design and population	CHW role	Findings
Carroll et al. (2007)	Boston and San Francisco, USA	Randomized controlled trial among older adults admitted for myocardial infarction (*n* = 93) or coronary artery bypass surgery (*n* = 154). Primary outcomes were rehospitalization and participation in a rehab program over 12 months.	Post-discharge, treatment group received home visit within 72 h of discharge and calls at 2, 6 and 12 weeks from advanced practitioner nurse. A peer advisor also made weekly calls for 12 weeks and were available for additional support by phone.	Intervention group had significantly higher overall participation in cardiac rehabilitation at 3, 6 and 12 months (*p* < .05), though increases in use over time was not significantly different between groups. Intervention group also had less hospitalizations at 3 and 6 months, though not statistically significant.
Crane-Okada et al. (2012)	California, USA	Randomized controlled trial among women > 50 years of age newly diagnosed and scheduled for surgery for stage 0–3 breast cancer (*n* = 142). Primary outcomes were psychosocial after 6 months.	3 post-surgical intervention groups with peer counselor telephone calls: Group 1—once per week for 5 weeks beginning 72 h post-surgery (immediate care); Group 2—once per week for 5 weeks beginning at 6-week post-surgery (delayed care); Group 3—by request (usual care).	Intervention (immediate or delayed) not associated with changes in perceived social support or anxious mood. Intervention was significantly associated with increased use of coping through seeking instrumental support (*p* < 0.05). Group without peer counseling had larger decrease over time of this coping strategy.
Hendren et al. (2011)	Rochester, USA	Descriptive qualitative study among newly diagnosed breast and colorectal patients (*n* = 103). Primary outcomes were need for patient navigation and navigation time spent by community health workers according to barriers to care.	CHWs worked as patient navigators to help with appointment reminders, coordinate care, insurance, logistical and social support, communication coaching, etc.	The most significant barriers to care were problems with medical communication, lack of social support, and medical insurance concerns. Higher need for patient navigation as measured by longer patient navigation times was associated with minority race/ethnicity, unemployment, and unmarried status.
Ivers et al. (2019)	New South Wales, Australia	Qualitative study using semi-structured interviews on the acceptability and accessibility of a pilot study for cancer care services with Aboriginal people with a cancer diagnosis or caring for someone with a cancer diagnosis (n = 79).	Multidisciplinary cancer care team (CCT) included an Aboriginal community health worker, counselor, nurse, and general practitioner to provide care coordination of every step from diagnosis, treatment, to post-op, including palliative care and grief support.	Most participants said the CCT improved access to care including adherence to clinic appointments, highly valued counseling and social support services, and that it was culturally appropriate.
Matousek et al. (2017)	Rural Haiti	Pre–post intervention study of a patient navigation intervention among rural Haitian communities served by Hospital Albert Schweitzer. Primary outcome was rate of elective surgery and signs and symptoms of surgical site infections.	Intervention involved two types of patient navigators—“community” navigators identified surgical patients, helped to navigate care, and 3 home visits within 1 month of discharge to evaluate for surgical site infections; “facility” navigators received patients at the hospital and navigate facility-based care.	Post patient navigation intervention, elective surgical operations significantly increased (1.92-fold increase, *p* = .017).
Matousek et al. (2015)	Rural Haiti	Descriptive pilot study of CHW program for post-operative home visits to detect surgical site infections (SSI) for 39 patients. Primary outcomes were on-time home visits, quality of SSI photographs, and agreement between surgeons and CHW on diagnosis.	5 CHWs conducted home visits to surgical patients within 30 days of discharge and took photographs of potential SSI using a smartphone application.	CHWs completed 95% of home visits and 92% of home visits on time. Using the application, CHWs took 117 photographs of potential SSI, of which 86% were deemed high quality. There was high agreement between surgeons and CHWs on diagnosis of SSI (85%).
Crane-Okada et al. (2010)	Santa Monica, USA	Qualitative study of the development and implementation of a CHW training program for breast cancer-specific topics, designed to support breast cancer survivors age 50 and older following breast cancer surgery. Primary outcomes were feasibility and efficacy of course including patient and participant satisfaction.	Volunteer CHW completed 10-week training course with content on breast cancer diagnosis, treatment options, psychosocial issues, resources specific to BCS, health information privacy, and mental health confidentiality.	High patient satisfaction and feedback reviews. Peer counselors also described experience as positive. Healthcare providers may benefit from learning how to best to utilize their volunteers' strengths and time, so that both are used most effectively.
Ennion et al. (2016)	KwaZulu Natal, South Africa	Qualitative study of persons with a lower limb amputation and the multi-disciplinary team (MDT) involved in prosthesis care. Primary outcomes were challenges of prosthesis care, referral patterns for pts who ultimately needed amputation, and defined roles for MTD members.	MDTs consist of surgeon, therapists, social worker, and prosthetists Baseline interviews were done with 3 prosthesis patients to establish existing challenges prosthesis care from patient perspective. Group discussions and semi-structured interviews were completed with other MDT members to identify challenges from care-giving perspective.	Patients are easily lost to care during process of undergoing amputation and receiving prosthesis. MDTs involved in prosthesis are often overwhelmed. Community CHWs may assist with relieving this workload but have unclearly defined roles and responsibilities. An interdisciplinary team with clearly defined roles may improve care.
Marais et al. (2005)	Western Cape, South Africa	Descriptive study of injuries occurring on selected farms within one year period, both occupational and other, needing some form of treatment. Primary outcomes were to describe the nature, extent, sources of injury, and to explore use of lay health workers (LHW) to document injuries and create injury database.	LHWs were part of a program where, usually a female (worker is chosen by other workers on the farm) is trained in the principles of first aid and general knowledge of a range of basic health issues, providing vital primary and immediate care to workers. LHWs were also trained in keeping records of injury and attended regular feedback and training sessions.	Most of the occupational injuries (60%) were treated by the LHWs or nurses on the farms showing LHWs acted as important primary source of care for trauma. The additional function of LHWs as documenting injuries makes important contributions to systematic, ongoing surveillance of farm injuries.
Sonderman et al. (2018)	Kirehe, Rwanda	Protocol for randomized control trial for post-operative surgical site infection (SSI) screening in women > 18 years who underwent cesarean section. Primary outcomes will be evaluation of CHW interventions on patient return to hospital for SSI, and feasibility of CHW intervention for post-operative follow-up.	Within post-operative day 10, a CHW will administer 10-question SSI screening protocol Arm 1: CHW will administer protocol at patient’s home, evaluate wound, and take photograph of wound Arm 2: CHW will administer protocol over the phone The control group (Arm 3) will not receive post-operative follow-up.	N/A

## Results

### Study characteristics

**Fig. 1:**
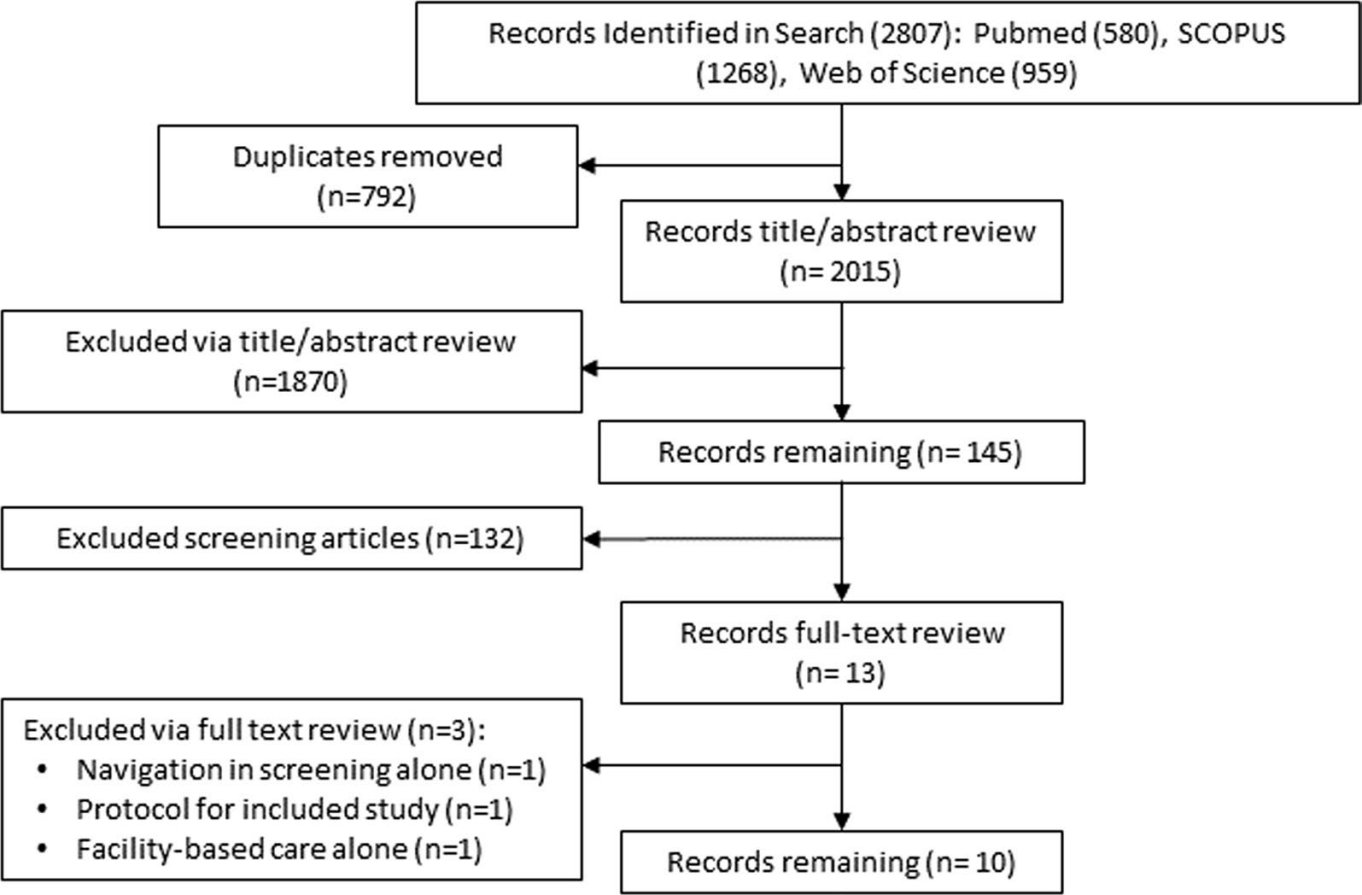
PRISMA search results

The search identified 2,806 results, 145 of which were found to be related to the 3-step surgical cascade, including screening, linkage to care, and post-operative care. Of these 145, ten studies within this group met our inclusion criteria and were included in our review (Fig. 1) [19, 27, 29–36]. Of the included studies, four studies were from the US, two from Haiti, two from South Africa, one from Rwanda, and one from Australia. Three were qualitative studies [32–35], two were randomized controlled trials (RCTs) [30, 31], two were descriptive studies [29, 36], one was a study protocol for a RCT [27], and one was a pre–post-intervention study [19] (Table 2).

Studies examining the CHW role in linkage to care and patient navigation involved care in general surgery [19], trauma [36] and oncology [32, 33]. Two of these four studies utilized interviews with patients or CHWs [32, 33], while the remaining two used quantitative data tracking causes of trauma in the community or increased utilization of surgical care. [19, 36] Those investigating CHW role in post-operative management involved general surgery [29], cardiothoracic surgery [31], obstetrics [27], orthopedics [35] and oncology [30, 34]. Of these six studies, four utilized patient or staff interviews [30, 31, 34, 35], and two assessed the presence of surgical site infections (SSI) [27, 29].

### Linkage to care & patient navigation

In each of the four studies involving linkage to care and patient navigation, CHWs served a unique vulnerable population. Three studies explored CHWs’ role in rural underserved communities—an aboriginal community in Australia [32], a rural farming community in South Africa, and a rural mountain community in Haiti [19]. In the remaining study, CHWs worked with vulnerable minority urban populations in the US [33].

The role of the CHWs in linkage to surgical care centered on addressing health disparities, isolating factors and vulnerabilities. One qualitative study [33] addressed racial disparities, one descriptive study [36], and one pre–post-intervention study [19] addressed geographical isolation, and one qualitative study addressed both [32]. The predominantly qualitative nature of these studies allowed for more granular identification of specific barriers in patient groups. However, many groups shared a particular dependence on CHWs for assistance with insurance enrollment and identifying sources of financial support [19, 32, 33, 36]. The CHW role in assisting with extra-hospital financial burdens, including arrangement of or transport to surgical clinic appointments, was particularly important for rural patients facing surgical deserts, which increased their geographical isolation from care and created higher financial costs related to transportation to care [19, 32, 36].

One descriptive study showed the potential role of CHWs in direct provision of trauma-related care in rural communities [36]. Within this study, CHWs were trained in the “principles of first aid and general knowledge of a range of basic health issues” [36]. This allowed CHWs to act as basic first responders to simple traumas occurring in a rural farming community, as well as linking patients to additional services if necessary [36]. While the majority of this study’s results described rates and causes of trauma reported, it provided CHWs with the additional role of record keeping and tracking the rates of trauma in their community. Their presence in assisting with establishment of a registry could help to guide future initiatives and legislation for their community, showing the CHW impact beyond direct clinical care alone [36].

Regarding racial and cultural barriers to care, two qualitative studies [32, 33] examined a wider role for CHWs in individualized patient navigation and maneuvering complex medical, cultural and social barriers through counselling, education, and connection to community resources. In doing so, CHWs were able to “level the playing field” for traditionally underserved surgical cancer patients [19, 32, 33]. Specifically, CHW interventions involved coaching in communication with medical providers, language translation, connection with social support, and even presence through sensitive periods, such as end-of-life care [32, 33]. CHWs accompaniment during care appointments allowed for immediate facilitation of patient challenges in communication and literacy, understanding health insurance paperwork, and better knowledge and trust in certain tests or treatments, barriers which are often enough to prevent patients from accessing appropriate care [19, 32, 33].

CHWs involvement during appointments also benefited healthcare teams. As one study noted, providers reported feeling reassured that CHWs would be able to help patients “get to where they needed to be” and increase adherence to their treatment plans [32]. This helped to allay the bias in providers’ perceptions of a patient’s ability to “handle” certain intensive treatments “due to their situations” [33] In addition, CHWs were able to assist the medical team as a mediator and advocate on important cultural issues. The significance of this role is reflected in one study’s finding of how medical teams lacking understanding of cultural habits or traditions were unable to provide care acceptable to patients [32]. Another study predicted that this cultural isolation would not only affect receipt of care, but also the doctor–patient relationship, patient adherence, and overall outcome [33]. CHWs were able to leverage their longitudinal relationships in sensitive circumstances, including guiding both patients and their families from the onset of diagnosis to beyond death and bereavement [32]. These intimate, long-term relationships were most valued by patients, and helped to encourage, and at times rebuild, trust in local healthcare systems [32].

Finally, CHWs were able to assist those patients isolated from the healthcare system due to stigmatized situations, including mental illness or homelessness by linking patients to counselling and social services [32]. CHWs also had a unique knowledge of community resources and connections which other providers may not know of, including patient support groups, food services, housing opportunities, or programs which provide subsidized or free medications [32]. One study noted the particular importance of such social support for patients despite inadequate recognition of this role within existing literature [33].

### Post-operative care

Six studies addressed the role of CHWs within post-operative care [27, 29–31, 34, 35]. Common needs within this phase of the surgical cascade included monitoring for SSIs, retaining patients in care, and surgical rehabilitation. Two studies explored the potential for CHWs to act as the initial diagnosticians for SSIs. Both studies took place in underserved areas, in which SSIs were noted to be a major contributor to poor surgical outcomes including sepsis, need for reoperation, increased healthcare costs, and death [27, 29]. Given the prevalence of cell phones and access to mobile networks in their communities, even in rural areas, both studies utilized digital mHealth technologies [27]. One descriptive pilot study introduced a smartphone application for CHWs which screened for SSIs through administration of a questionnaire and pictures of the incision site taken during a post-operative home-visit occurring within 30 days of discharge [29]. CHWs accurately diagnosed SSIs up to 85% of the time, as matched to physician diagnosis, and were able to complete 30-day follow-up visits with over 90% of post-operative patients [29]. Building on the previous study, the second study was a protocol for an ongoing RCT that seeks to address SSIs in women following a cesarean section [27]. This study is similar to the first in its assessment for SSIs with an app-based questionnaire and photograph of the incision site, but seeks to expand through comparing CHW in-person intervention at a patient’s home with a fully virtual CHW assessment over the phone [27].

CHWs are also seen to have roles in improving rates of patient rehabilitation post-operatively. Two RCTs explore the role of CHWs following cardiac or breast cancer surgeries, with particular attention to older patients more vulnerable to poor outcomes [30, 31]. It was notable that these studies approached rehabilitation in different ways, addressing both the physical and psychosocial aspects. One RCT paired patients with both an advanced nurse practitioner (NP) and a peer advisor who previously completed the rehabilitation program and actively participated in a healthy lifestyle [31]. The patient received a post-discharge home visit from the NP and weekly phone check-ins from the peer advisor, addressing patient concerns, dispelling inaccurate expectations, and encouraging patients to complete the rehabilitation program. While this study found that patients within the intervention group had higher overall use of post-operative rehabilitation at 3, 6, and 12 months following discharge, the rate of change in participation the rehabilitation program over time did not differ between groups.

Another RCT focused on the psychosocial aspect of rehabilitation, particularly following life-altering events such as a cancer diagnosis or a large surgical procedure [30]. This study utilized trained, senior peer counselors to contact patients through telephone check-in’s following a recent procedure for breast cancer [30]. This study identified this acute post-operative period as a particularly vulnerable time during which family and friends may return to their normal routines after supporting a patient through diagnosis and surgery, leaving some patients alone [30]. Patients were randomized to post-surgical calls with the peer counselor beginning either immediately post-operative, 6 weeks following, or upon request. The groups assigned to receive calls were found to have increased use of coping by seeking instrumental support, or “getting advice or help from other people about what to do” [30]. Patients assigned to CHWs were also noted to have longer use of this coping strategy over time compared to patients who had to request calls with CHWs. Anxiety or perceived social support did not differ between groups and tended to decrease over time.

Overall, both studies noted how CHWs may address the particular vulnerability of older patients within rehabilitation following surgery, including risks for greater mood disturbance, poor coping strategies, or limited social support [30]. However, they also noted the limitations associated with elective participation in interventions, as patients who chose to participate in CHW interventions were likely more open to social interaction and support at baseline [30, 31].

Finally, two studies reviewed the role of CHWs in post-operative teams [34, 35]. One qualitative study addressed the training of CHWs as phone counsellors for breast cancer survivors [34]. CHWs completed a 10-week training course with content on breast cancer diagnosis, treatment options, psychosocial issues, and resources specific to breast cancer survivors, which they were able to utilize during phone counselling sessions with patients [34]. Participating CHWs found that their roles not only included providing patients with encouragement and affirmational support, but also recognizing indicators of poor coping and encouraging patients to contact their care team regarding their symptoms or concerns [34]. Thus, beyond social support, CHWs acted as important influences for care-seeking upon early signs of need [34]. The second qualitative study examined challenges associated with post-amputation prosthesis care and the importance of an inter-disciplinary team [35]. CHWs once again showed promise as resources to help decentralize health care and retain patients in care, helping them to complete the journey from amputation to prosthesis [35]. However, this study noted the unclear roles of CHWs, which limited their effectiveness, and emphasized the need for health provider collaboration to ensure appropriate patient care through the varying steps of prosthesis care [35]. Both studies addressed the important idea that surgery is not the last step in care, as significant choices about rehabilitation or future adjuvant treatments often remain [34]. Having CHWs as a part of an inter-disciplinary team helping to guide post-operative care and decisions remain critical.

### Challenges in CHW integration

Studies reported several challenges associated with CHW interventions in the surgical cascade. While most studies specifically selected CHWs based on their connection to the specific community served, [19, 27, 29, 31, 32, 35, 36] those which did not reported this as a weakness. Specifically, the strength of partnerships between CHWs and patients varied, thus potentially impacting the level of benefit attained by patients overall [30, 31, 33]. A lack of clarity in the CHW role was another weakness reported to have impacted the quality of care in some studies [34, 35]. One study reported how CHWs frequently felt helpless to assist with patients’ concerns regarding their treatment or side-effect profiles [34], while another study reported how CHWs were overwhelmed with the range of responsibilities expected of them [35]. Multiple studies noted the importance of healthcare providers learning how to best utilize the strength of CHWs [34, 35]. Finally, CHW attrition rates were recognized as a weakness within CHW interventions. One study noted frequent high turnover rates of CHWs due to poorly defined roles, lack of training, and low remuneration [35]. Another study involving CHW training noted a 50% attrition rate prior to the completion of training, despite both patients and CHWs participating in the study reporting the experience to be rewarding and beneficial [34].

## Discussion

A misconception reigns that surgery is an intrinsically unaffordable luxury for a large portion of people; however, increasing access to essential surgeries is not only possible but highly cost-effective in life years or disability-adjusted life-years (DALYs) gained [37]. To address the myriad of challenges to increasing access to quality surgical care, it is critical to view surgery as a cascade of events rather than one single clinical event. Our review shows evidence that CHWs can play a valuable role through the entirety of the surgical cascade, addressing complex barriers to care associated with patient vulnerabilities and improving outcomes of surgical patients overall. While the literature is still nascent, CHWs may promote timely presentation for surgeries, appropriate adherence to post-operative care, and decreased rates of SSIs, decreasing poor post-operative outcomes and maximizing surgical benefit.

Through this review, numerous unique strengths of CHWs were identified in linking patients to surgical care and promoting post-operative care. Multiple studies in our review showed how patients most valued the personalized and longitudinal relationships with CHWs, something which is difficult to obtain from surgeons who are limited by their clinical and operative demands [26, 29, 30]. The post-operative period may result in the fading away of health providers which were intimately connected with patient care. In patients facing varying degrees of isolation, their vulnerabilities may be exacerbated during this time [30]. CHWs are able to spend more time directly interacting with patients than many other healthcare workers, particularly in community or non-facility settings, gaining a deep understanding of the patient and their complex needs, and establishing intimate, longitudinal relationships, acting as counselors and peers alike [31, 34, 35]. Studies have also demonstrated the capacity of CHWs to manage time-intensive responsibilities, including arrangement of external resources or counselling patients and family, which other surgical team members often are unable to assume due to time constraints [33]. Being based in the community, CHWs also have the ability to decentralize surgical care and bring care opportunities to patients directly.

One frequently proposed area of improvement was the need to identify how CHWs would best fit into the surgical team. In 2008, the WHO proposed 115 tasks out of 313 essential tasks overall which may be performed by CHWs with regard to HIV treatment [38]. Of these, 48 tasks were related to medical skills, while 67 tasks were socially oriented, involving counselling, education, or support [38, 39]. Our review reflected the unique spectrum of CHW skills required in the surgical cascade, ranging from interventions addressing complex social issues to clinical assessment for the presence of SSIs. This spectrum of responsibilities not only places an excessive workload on CHWs, but also can force CHWs to work beyond the scope of their training [40]. CHWs should not be viewed as just ‘another pair of hands’ or as fillers for insufficient clinical staff [35, 38, 40]. Particularly within a surgical team, where each member has a designated role to play, clear and thoughtful designation of CHW roles and responsibilities is critical. The WHO guideline on health policy and system support to optimize community health work programs provides guidance for international standards for the creation and management of CHW programs, but there is no guidance specific to surgically related responsibilities and training and how they can be adapted to fit local health systems, workforces, and population needs [41]. Establishing a similar set of essential tasks for surgical care would help to guide training efforts and appropriate supervision to assure the quality of CHW interventions [35, 38].

It is also important to note that despite positive feedback from both CHWs and patients regarding participation in CHW interventions, attrition rates for CHWs remain high [38, 40]. The uniquely situated strength of CHWs in creating close and trusting relationships with patients also requires high demand in time and resources by CHW programs to support such dedicated, longitudinal commitments to patients. Challenges of costs for adequate training and funding such a cadre of workers have been noted by multiple CHW programs [19, 32, 33]. While formal incentives for CHWs vary by programs within the field, ranging from salaries, worker recognition, or career advancement opportunities, one of most effective protective factors against attrition is success in the role of a CHW [38]. One systematic review noted how training increases the likelihood of CHW success by equipping them with the appropriate knowledge and skills to become a trusted resource for their community [17]. Similar trends exist within supervision, where adequate supervisory support and buy-in from other members of the health system increases CHW legitimacy within the community, and thus increases the likelihood of success [17, 40, 42, 43]. As shown in our review, the roles for CHWs within the surgical cascade may be wide-ranging, thus requiring training for both technical and social competency [17]. Investment in appropriate training for CHWs will ultimately promote the long-term success of integrating CHWs into the surgical cascade.

Our review is limited by several factors. A disproportionate number of studies addressed the first step of the surgical cascade, screening, which is why we chose to omit literature related to this stage in this review. The remaining studies we included addressing linkage to care and follow-up care are few and mostly preliminary. Of included articles, quality was variable due to small sample sizes or predominantly descriptive study designs. In addition, CHWs played multiple roles in all aspects of patient care in many of these studies, complicating the ability to establish a direct relationship between CHW involvement and improved patient outcome. However, the qualitative methodology used in several studies helped to elucidate details about CHW strengths and weaknesses in surgical care and should encourage future studies [44].

The global nature of this review resulted in a diverse array of care settings and populations, limiting generalizability. However, the general trends of CHW effectiveness across these settings suggest that CHWs may be beneficial to the surgical cascade in HICs and LMICs alike [17]. Finally, our search was limited to studies published after 2000 as we aimed to capture the recent literature regarding CHWs in the surgical cascade. While the paucity of existing data suggests this is a growing field, it is possible that we did not capture relevant studies prior to 2000. In future studies of CHWs in the surgical cascade, it is important to encourage research of CHW impact on surgical patient navigation and post-operative management, much like what already exists for patient screening.

While this review has shown multiple examples of how CHWs were utilized to overcome individual barriers, there are many compounding systemic barriers that prevent patients from accessing affordable and quality surgical care. The full extent of these barriers is beyond the scope of this review, but key issues mentioned in existing literature include insufficient numbers of healthcare provider and surgeons, inadequate training resulting in low surgical trainee pass-rates on licensing exams, dysfunctional healthcare referral networks, or lack of investment in health systems [10, 45–47]. As one study stated, inadequate access to care is more than a CHW problem, it is a systems problem [35]. While our review supports how CHWs can strengthen surgical teams, access to care, and patient outcomes, there must also be a strong existing foundation of adequate surgical staff and facilities alongside affordable and accessible healthcare.

## Conclusion

As representatives of their communities, CHWs can act as powerful advocates for underserved populations. This is a particularly critical role in the surgical care, which remains one of the most limited health services for many around the world [19]. Although a majority of surgical care remains hospital and institution-based, the complexities of surgical care regarding both individual disease processes and navigation of the healthcare system emphasize the need for viewing surgical care as a cascade of events rather than a singular operative event. The flexible and broad ranging skillset of CHWs can play an integral role in helping patients in every phase of the surgical cascade. Research within the area of CHW intervention in the surgical cascade is difficult given multifaceted interventions and results which are often affected by multiple variables. However, clear, concise methods of measuring results is needed to build a body of comparable, high-quality literature addressing topics including clear integration of CHWs into the surgical team, quality of CHW intervention, and sustainability of the intervention in the future. Overall, surgery progresses leaps and bounds every year, but the most curative of procedures means little if those in need are unable to access it. Thus, establishing strong relationships with the communities served must be a critical part of what surgeons and surgical teams do. CHWs offer an effective and powerful resource by which to achieve these goals.

## Data Availability

Not applicable.
